# A Case of Poisoning by Tartar Emetic

**Published:** 1848-01

**Authors:** John T. Gleaves

**Affiliations:** Green Hill, Tennessee


ART. III.—*A Case of Poisoning by Tartar Emetic.* By JOHN T. GLEAVES, M.D., of Green Hill, Tennessee.

On the 27th of October, 1847, I was called to see R., a young man of good constitution and temperate habits, aged about twenty-four years. The messenger informed me that he had left the patient an hour before pulseless, speechless, and to all appearance in a dying condition. I found him on my arrival lying on his back, breathing slowly and laboriously, his face pale and altered, features shrunken, eyes fixed and turned upwards, pupils dilated, surface cold. He appeared to be unconscious, but stated afterwards that he knew what was passing around him, but was unable to speak. The action of his heart was intermitting and extremely feeble, and no pulse could be felt at the wrists.

On inquiring into the history of the sudden illness, I learned that R. had taken a dose of tartar emetic, and that the quantity actually swallowed by him was a *table spoonful.* This he did at three o'clock, in the afternoon, and an hour and a half afterwards, although he had drunk freely of warm water and tickled his fauces repeatedly with his fingers, no vomiting had occurred. Altogether, he vomited for the first three hours only two or three times, and the matter ejected was chiefly the warm water taken to favor the emesis. About two hours after he had swallowed the medicine, he felt an inclination to evacuate his bowels, and going into the yard for that purpose, found himself unable to return to the house. He was carried in and laid on a pallet before the fire. The alvine discharges continued, and I found him passing involuntarily liquid stools in great excess. The thin matter thus discharged had actually run from one end of the room to the other.

I ordered immediately laudanum, in a decoction of galls, by the mouth, and in the shape of injections; applied sinapisms to the spine, abdomen and extremities, and directed brandy toddy to be given liberally.

This course was adopted at six o'clock, three hours after the poison was taken. In about seven hours the purging ceased, and reaction was established; the patient was able to give a rational answer to questions, and to describe his sufferings. He complained of great thirst, and a sense of burning in the fauces, œsophagus, stomach and lower bowels. Applied a blistering plaster to the abdomen, and for the laudanum and gall nuts substituted coffee. Stomach grew extremely irritable; vomitings repeated, matter discharged being tinged with blood; tongue red and smooth. Directed leeches to be applied to the epigastrium; gum water; morphia and calomel in minute doses.

I left the patient at day light with pulse 120, quick and small, stomach painful on pressure, and ejecting every thing taken.

22d. At five o'clock, P. M., I found my patient again cold, pulseless and speechless; abdomen tympanitic, and painful to the touch. The purging was arrested, but vomiting had continued through the day. Friction with flannels wet with warm spts. turpentine; sinapisms over all the body not blistered; flannel rollers saturated with spts. turp., to the extremities; hot applications to the feet. Reaction soon followed, and in an hour the condition of things was more promising than in the morning. Patient slept half an hour quietly; on waking, vomited a glairy matter mixed with blood. Mucilaginous drinks to be continued, with occasional small doses of morphia and sulph. quinine. Bowels to be moved by olive oil.

23d. At four o'clock, P. M., learned that my patient had rested well the night before. Still vomits occasionally; bowels have acted; the passages dark, offensive, and composed in part of grumous blood. Complains of sore throat and difficulty in deglutition. On examination, find his fauces covered with pustules, some of which, having discharged their matter, have left small superficial ulcers. Pustules around the blistered surface on the abdomen. Stomach and bowels still tender to the touch, but no tympanitis. Painful micturition; the urine copious and high colored.—Morphia, quinine, and mucilaginous drinks; diet, gruel or chicken broth. I left him at nine o'clock asleep, skin moist and warm, pulse soft and about 100; breathing improved.

24th. Patient improving; the whole surface of his body and neck studded with genuine *tartar emetic pustules.* Complains of no pain, except the burning and itching of the skin from the pustules.

25th. Patient has rested well since last visit. Alvine dejections still slightly tinged with blood. Pustules appearing on the extremities.

27th. Pustules on the body are healing, while those on the extremities are proceeding to maturity. Burning sensation very distressing. Some of the pustules on the body are as large as a plum, and the matter so deep-seated in some, as to require an incision to discharge it.

The patient went on steadily to improve, and the process of desquamation was completed about the end of the second week from the time of the appearance of the pustules. He is now in as perfect health as he enjoyed before taking the poison.

Since treating the above case, I have witnessed pustulation of the surface in the case of a pneumonic patient, to whom I administered freely the tartar emetic.

APRIL 20, 1847.

